# Hyperidrosis

**Published:** 1883-10

**Authors:** 


### HYPERIDROSIS.

### EXCESSIVE SWEATING OF THE HANDS.

For this annoying condition, Dr. F. H. Alderson says in the *Lancet*, July 28th, 1883 :

The patient should soak his hands night and morning in warm water, in which should be dissolved about two drachms or half an ounce of the chloride of ammonium, and about twice as much carbonate of soda (crystals), enough water to be used to well cover the hands. I generally prescribe for my patients sufficient for six applications ; and as skins vary in tenderness, tell them to use as much as will temporarily to a slight extent cause the wrinkling known as *cutis anserina*, a condition which I describe to them as looking like the hands of a washerwoman. After well bathing, the hands are to be rubbed with the following embrocation : Tincture of iodine one drachm, compound camphor liniment and glycerine, of each a drachm and a half, and compound liniment of belladona one ounce. (If for the hands, a drachm of eau de cologne makes the embrocation more agreeable.) The embrocation to be applied twice a day. A cure quickly follows. This treatment is equally appropriate and successful for excessive sweating and even bad smelling feet, for that odor is due to the excessive function of the sudoripharous glands.

